# Ride-Hailing Services and Alcohol Consumption: Longitudinal Analysis

**DOI:** 10.2196/15402

**Published:** 2021-01-27

**Authors:** Gordon Burtch, Brad N Greenwood, Jeffrey S McCullough

**Affiliations:** 1 Carlson School University of Minnesota Minneapolis, MN United States; 2 Department of Information Systems and Operations Management George Mason University Fairfax, VA United States; 3 Department of Health Management and Policy University of Michigan Ann Arbor, MI United States

**Keywords:** binge drinking, drunk driving, road traffic safety, ride-hailing, alcohol consumption, difference in differences, Uber

## Abstract

**Background:**

Alcohol consumption is associated with a wide range of adverse health consequences and a leading cause of preventable deaths. Ride-hailing services such as Uber have been found to prevent alcohol-related motor vehicle fatalities. These services may, however, facilitate alcohol consumption generally and binge drinking in particular.

**Objective:**

The goal of the research is to measure the impact of ride-hailing services on the extent and intensity of alcohol consumption. We allow these associations to depend on population density as the use of ride-hailing services varies across markets.

**Methods:**

We exploit the phased rollout of the ride-hailing platform Uber using a difference-in-differences approach. We use this variation to measure changes in alcohol consumption among a local population following Uber’s entry. Data are drawn from Uber press releases to capture platform entry and the Behavioral Risk Factor Surveillance Systems (BRFSS) Annual Survey to measure alcohol consumption in 113 metropolitan areas. Models are estimated using fixed-effects Poisson regression. Pre- and postentry trends are used to validate this approach.

**Results:**

Ride-hailing has no association with the extent of alcohol consumption in high (0.61 [95% CI –0.05% to 1.28%]) or low (0.61 [95% CI –0.05% to 1.28%]) density markets, but is associated with increases in the binge drinking rate in high-density markets (0.71 [95% CI 0.13% to 1.29%]). This corresponds to a 4% increase in binge drinking within a Metropolitan Statistical Area.

**Conclusions:**

Ride-hailing services are associated with an increase in binge drinking, which has been associated with a wide array of adverse health outcomes. Drunk driving rates have fallen for more than a decade, while binge drinking continues to climb. Both trends may be accelerated by ride-hailing services. This suggests that health information messaging should increase emphasis on the direct dangers of alcohol consumption and binge drinking.

## Introduction

### Background

Alcohol consumption is a serious public health issue with significant implications for personal health and well-being [1]. Between 2006 and 2010, more than 88,000 people lost their lives as a result of alcohol abuse in the United States; 56% of those deaths occurred because of acute events like motor vehicle collisions or alcohol poisoning [2]. Scholars have tied alcohol consumption to a host of deleterious societal outcomes, including spousal and child abuse [3]; inability to maintain gainful employment [4]; and personal health concerns in the form of diabetes, liver disease, and sexual dysfunction [5]. Prior work has also documented many associated factors that can accelerate alcohol consumption, including peer pressure and social norms [6], financial distress [7], and mobility and public transit accessibility [8], the context of this investigation.

Urban mobility is particularly notable when one considers the changes in the market for individual transportation over the last 10 years (ie, ride-hailing). Whereas people were once forced to drive themselves or depend on often unreliable alternatives (eg, public transit or taxis), ride-hailing services now offer a simple solution that is integrated with an individual’s smartphone. Uber, for example, completed its 10 billionth ride in June 2018, and ride-hailing more generally accounts for a significant share of the urban mobility market. Moreover, recent academic work has begun to assess the relationships between ride-hailing services and public health outcomes (eg, traffic fatalities [9,10], occurrence of assault [11-13], ambulance use [14], and rates of drunk driving [15]). In this work, we delve into the question of whether ride-hailing services affect the extent of alcohol consumption within the population (ie, prevalence) as well as the intensity of consumption (binge drinking).

### Research Objectives

Research into the social implications of ride-hailing services is wide ranging. A significant body of work has been devoted to the economic implications of ride-hailing, including the effect on entrepreneurship [16], durable goods purchases [17], and the labor market more broadly [18]. Research has also begun to examine social issues stemming from ride-sharing. Findings are diverse and include diminished rates of sexual assault [12], increases in property crimes [11], and a reemergence of bias based on ascriptive characteristics which has traditionally be absent from online transactions [19,20]. However, the largest body of work in this space has, unsurprisingly, focused on motor vehicle safety [9-11] and, more specifically, the effect on drunk driving [13,15].

The potential drunk driving and ride-hailing relationship is intuitive. Platforms like Uber are more accessible than limousine services and more ubiquitous and reliable than taxis [21,22], and thus people may shift from driving under the influence to ride-hailing. The fact that ride-hailing is often a complement to public transportation [23,24] only underscores this idea. Because ride-hailing services can solve the last mile problem for people reliant on public transportation and provide point-to-point transportation for those not reliant on public transportation, the need to drive should be diminished.

We build on past lines of inquiry and offer a first consideration of whether ride-hailing services facilitate a greater extent of alcohol consumption (a larger number of individuals consuming alcohol) and a greater intensity of alcohol consumption (more binge drinking). Ride-hailing services might simply displace alcohol consumption which used to occur at home by making it easier to go to bars, restaurants, and night clubs. However, past work has found that people elevate their alcohol consumption in the presence of an assured transportation option (eg, a designated driver [25]). For example, when the city of Washington, DC, extended the Washington Metropolitan Area Transit Authority train hours (ie, mass transit) to after last call, there was not only a sharp drop in drunk driving but a significant increase in property crimes associated with drinking to excess [8]. Given the relative ease with which individuals can obtain a ride-hailing trip following a night of drinking, it is plausible that ride-hailing services would increase both the extent and intensity of alcohol consumption. Coupled with the public costs associated with excessive alcohol consumption, approximately $250 billion in the United States in 2010 [26], any connection between ride-hailing access and drinking activity would have important policy implications.

Our analyses test two hypotheses. Our first hypothesis (H1) is that access to ride-hailing services increases alcohol consumption at the extensive margin (ie, raising the number of individuals drinking alcohol). Our second hypothesis (H2) is that access to ride-hailing services increases alcohol consumption at the intensive margin (ie, the amount of alcohol a given individual consumes in a sitting). The distinction between these hypotheses is important. While public health interventions and many laws focus on the intensity of individual alcohol consumption (drinking to excess), recent work suggests that the extent of alcohol consumption may also be a concern in its own right as even one drink of alcohol daily has been associated with negative health outcomes [1].

Considering our two hypotheses, it is also important to note previous research, which suggests that the effects of ride-hailing services are heterogeneous across markets [23,24]. Ride-hailing platforms are subject to network effects (riders require a sufficient supply of drivers and vice versa), thus population density is typically crucial to their adoption. Further, past work notes that ride-hailing services are often employed as complements to public transit [23], and thus their relationship to drinking activity is likely to be more apparent in locations characterized by higher population density, where transit options are more readily available (eg, taking the train into the city and an Uber home). Finally, locations characterized by greater population density are also more likely to bear a greater density of drinking establishments, suggesting a larger upper bound on the possible rise in drinking. Given these points, we conduct our analyses while distinguishing between locations characterized by higher versus lower population density.

## Methods

### Data

We combine data on the diffusion of ride-hailing services with information on alcohol consumption and population density. Data on alcohol consumption are drawn from the Behavioral Risk Factors Surveillance Survey (BRFSS). The BRFSS data are collected as part of the Selected Metropolitan Area Risk Trends program from the Centers for Disease Control and Prevention (CDC). These data include annual counts of respondent answers to questions about risky behaviors, including alcohol consumption, for a select set of metropolitan areas. We examine two such questions: respondents’ engaging in any drinking in the prior 30 days and binge drinking in the past week (the latter defined as consuming 5 or more drinks in a single session for males and 4 or more drinks for females). BRFSS measures for any and binge drinking are not available for every Metropolitan Statistical Area (MSA) year because the survey is conducted by the CDC only in those locations that meet certain sampling criteria, and positive response counts (responses of yes) are reported only in cases where a minimum threshold of 50 responses was obtained for the MSA. As a result, some locations enter and exit the sample over time. That said, numerous studies have been conducted that speak to the validity and representativeness of the BRFSS data.

Data on Uber’s entry into different locations are drawn from public press releases and announcements on Uber’s website (see Multimedia Appendix 1 for a list of Uber entry dates for the geographies comprising our sample). Uber presence is coded as a dichotomous indicator equal to 1 once Uber (in any format, whether Uber X or Uber Black) enters any city or township within a given MSA, and 0 in preceding years. We focus our analysis on Uber for 3 reasons. First, competing services (eg, Lyft) systematically enter after Uber, except in a handful of rare instances. Second, Uber is the largest ride-hailing platform by rider base and revenue ($82.4 billion in 2019) [27]. Third, the other dominant ride-hailing service in the United States, Lyft, operates a very similar business model, implying it would have a very similar effect. In fact, they are so similar that drivers and riders frequently multihome, switching between the two services [28].

Finally, we incorporate US Census Bureau measures of MSA population density, because prior work has documented that Uber’s influence is particularly pronounced in dense urban areas [15]. Markets with dense populations are thicker—having more potential drivers, consumers, and destinations within a region—and it is reasonable to expect that consumers experience better service with reduced wait times and lower fares. As such, we expect larger associations in these population dense areas.

Our sample spans 2010 through 2016, comprising 113 MSAs in which both ride-hailing entry dates were recoverable, and where BRFSS measures of alcohol consumption were available for at least 2 years. Each MSA is observed for an average of 6.5 years. This combined sample, coupled with the fact that Uber enters different locations at different times, allows us to measure systematic changes in alcohol consumption after ride-hailing service entry. Summary statistics are in Table 1. As can be seen in the table, any drinking is reported by 56.12% (1025.78/1827.91) of respondents on average, while 17.23% (315.03/1827.91) report binge drinking. Uber first enters our sample in 2011. Entry was gradual for the first few years, accelerated rapidly in 2014, and was nearly universal by 2016.

**Table 1:** Descriptive statistics.

Variable	Mean (SD)	Median	Minimum	Maximum	Observations
Uber presence	0.41 (0.49)	0	0	1	730
Binge drinking^a^	315.03 (311.67)	205	18	2067	728
Any drinking^b^	1025.78 (1016.11)	675	79	6030	730
BRFSS responses^c^	1827.91 (1682.07)	1293.5	464	9333	730
Population density^d^	2469.02 (2191.48)	1953.5	7.2	13,597.50	588

^a^Binge drinking reflect the number of respondents who indicated they had 5 or more drinks (in the case of males; 4 or more drinks in the case of females,)on a single occasion in the prior 30 days.

^b^Any drinking reflects the number of respondents indicating they consumed at least one alcoholic beverage in the prior 30 days.

^c^BRFSS: Behavioral Risk Factors Surveillance Survey.

^d^Population density reflects people per square mile within 10 miles of city hall.

### Statistical Analysis

Measuring the relationship between alcohol consumption and ride-hailing service availability is inherently challenging, particularly given that Uber may have selectively entered markets characterized by heavy—or even growing—rates of alcohol consumption in an effort to optimize both its ridership and profits. That is, ride-hailing services are known to be used more intensely on Friday and Saturday nights, and Uber may have selectively entered markets with higher levels of alcohol consumption with the objective of capturing that demand. We address this selection problem by employing a difference-in-differences approach [29,30]. In doing so, we measure the change in alcohol consumption before and after Uber entered into an MSA relative to the change in markets where Uber had yet to enter. Parameters are estimated based on a difference-in-differences Poisson regression, incorporating location (*α_l_*) and time (*τ_t_*) fixed effects, with standard errors clustered by MSA. Equation 1 reflects the regression model that we estimate. Our independent variable of interest is *Uber*, which takes a value of 1 if MSA *l* had received any form of Uber as of year *t*. Our coefficient of interest is *β*, which reflects our estimate of the relationship between Uber entry and alcohol consumption. Additionally, we incorporate BRFSS respondent counts for MSA *l* as an exposure term in the regression.

*Log* (*Drinking_l,t_*) ~ *α_l_* + *τ_t_* + *β⋅Uber_l,t_* + *ε_l,t_* (1)

We calculate and report the estimated change in the incidence rate of any drinking and binge drinking among respondents in a location based on marginal effects. Further, given that ride-hailing’s impact should differ across markets based on population density, we split the sample and estimate separate models for markets having above- and below-median population densities, based on measures reported by the US Census Bureau, derived from the 2010 Census.

### Robustness and Sensitivity

Before examining the results of the above estimations, we first discuss the robustness and sensitivity checks. First, we began by testing for pre-entry changes in alcohol consumption in the periods leading up to Uber’s entry into an MSA. Significant differences prior to Uber’s arrival would imply violation of the parallel trend assumption that underlies difference-in-differences estimation. Evaluating the parallel trends assumption is important to ensure our estimates are not driven by Uber systematically selecting into cities, in a manner that merely correlates with expected or ongoing growth in drinking activity. As Uber representatives have emphasized its widespread use in traveling to or from bars on weekend nights, selection effects on Uber’s part are certainly possible and of concern. Put another way, while the location fixed effects included in our regression model make it robust to Uber entering markets with higher persistent levels of drinking activity or any other stable and unchanging features of that location, systematic entry by Uber into markets that exhibit growing trends in binge drinking, even before Uber’s arrival, would violate the baseline assumptions of the model by creating a dynamic, time-varying, confounder for the estimated association between Uber presence and alcohol consumption.

To implement this test, we estimate a relative time variant of difference-in-differences regression [30]. In doing so, we construct a set of indicators capturing the relative (to Uber entry) year that an observation had taken place, which is defined based on the timing of Uber’s arrival in a particular MSA. In this regression, we omit the 2 years preceding Uber’s arrival, taking these as a joint reference period. It should be noted that a degree of freedom is lost in the estimation because we jointly estimate both absolute and relative time dummies (ie, year and year from Uber entry), so two reference periods must be omitted for the other model parameters to be identified. This specification allows us to estimate the relationship between Uber entry and drinking flexibly, as we can observe differences (or the absence of difference) in both pre- and postentry drinking trends. With this regression, if the assumptions of the model hold, we expect to observe positive and significant differences in the years following Uber’s arrival relative to the 2 years just prior to an Uber arrival in any MSA but no significant differences in the years prior (eg, 3, 4, or 5 years prior).

Second, we explored sensitivity of our results to our choice of population density measure. Our baseline analyses employ a measure from the Census Bureau reflecting the population per square mile residing within 10 miles of city hall. Given that this choice of radius is to some degree arbitrary, our goal is to ensure that any results are not sensitive to the choice. We therefore also considered splits using alternative measures of population per square mile, namely with a radius of 5 miles to city hall and again within 2 miles of city hall.

## Results

### Statistical Analysis

Results of our primary regression analyses appear in Table 2. We observe no significant relationship between Uber’s presence and the extent of alcohol consumption (ie, any drinking). Table 2 indicate a lack of significant change in any drinking across both high- and low-density markets. Uber’s entry is, however, associated with a significant rise in binge drinking within high-density MSAs. Table 2 reports a marginal estimate of 0.71 (95% CI 0.133% to 1.289%). This indicates that Uber’s entry is associated with an additional ~0.70% increase in the population’s binge drinking rate. This corresponds to a 4% relative increase in the binge drinking population in a given MSA.

**Table 2:** Marginal effects of ride-hailing on alcohol consumption in high- and low-density Metropolitan Statistical Areas.

Characteristic		Uber presence % (SE)	95% CI	Year fixed effects	MSA^a^ fixed effects	Observations	MSAs	χ^2^
**Any drinking**								
	Low density	–1.10 (0.63)	–2.33 to 0.13	Yes	Yes	264	61	64.99 (7)
	High density	0.61 (0.34)	–0.05 to 1.28	Yes	Yes	324	52	120.15 (7)
**Binge drinking**								
	Low density	–0.12 (0.61)	–1.31 to 1.07	Yes	Yes	263	61	700.57 (7)
	High density	0.71 (0.30)	0.13 to 1.29	Yes	Yes	324	52	265.65 (7)

^b^MSA: Metropolitan Statistical Area.

It should be noted that the estimated association between Uber presence and binge drinking in high-density MSAs persists following a correction for multiple comparisons. Specifically, applying a very conservative Bonferroni correction, we would rely on a significance threshold of 0.025 (for a 1-tailed test, consistent with the directional nature of the hypotheses). The observed *P* value associated with the relationship between Uber presence and binge drinking within high-density MSAs is .02. Although we observe a statistically significant association between Uber presence and binge drinking in high-density MSAs, it is important to note that the estimated coefficient is not significantly different from the estimate recovered in low-density MSAs. Thus, our findings only support a conclusion that Uber is significantly associated with a rise in binge drinking in high-density MSAs, not that the association is systematically larger in high-density MSAs as compared with low-density MSAs [31].

### Robustness and Sensitivity Results

**Figure 1:**
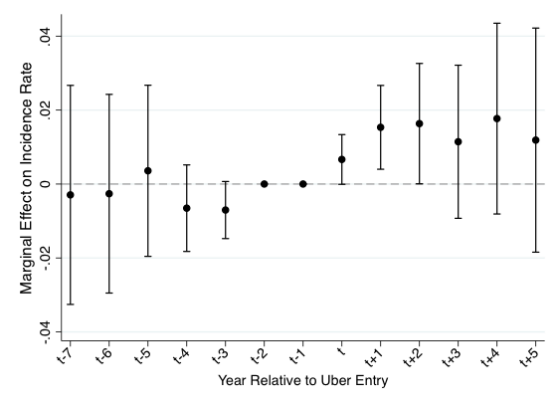
Marginal effects of relative year dummies in dense Metropolitan Statistical Areas (90% confidence intervals).

Figure 1 provides a graphical representation of relative time differences for binge drinking in high-density MSAs. As discussed, we employed this model to test for time-varying differences in binge drinking preceding Uber’s entry, which would indicate a possible spurious relationship. Each point is a parameter estimate corresponding to leading (t–7 to t–3) and lagged (t to t+5) differences. Consistent with prior work, all estimates are conducted relative to the 2 years (t–1 and t–2) preceding Uber’s entry (period t) [15]. The leading, or preperiod, estimates are uniformly small and statistically insignificant despite our use of relatively conservative (90%) confidence intervals. These results provide empirical support for our difference-in-differences strategy as they suggest no violation of the parallel trends assumption. It therefore appears reasonable for us to believe that Uber’s entry is exogenous with respect to drinking activity, conditional upon controls. The significant association with binge drinking persists in years following Uber’s entry into a market (ie, t+1 to t+5). These findings are again consistent with our difference in differences strategy. As expected, the confidence intervals are wider in the tails of our distribution (eg, for t–4 or less and t+3 or more) where there is less data available to perform the estimation (ie, there is a lack of power in the estimations because few cities have had Uber for such a lengthy period of time). In sum, our findings are consistent with those reported in Table 2 and provide empirical support for the assumptions of our research design (ie, parallel trends).

Results of our sensitivity around the population density measure are in Table 3 (ie, population per square mile within 5 miles and 2 miles of city hall). With each alternative measure, we continue to observe that higher density MSAs exhibit a significant increase in the incidence of binge drinking following Uber’s arrival. No significant association is observed in low density MSAs.

**Table 3:** Robustness check: marginal binge drinking effects using alternative density measures.

Characteristic		Uber presence % (SE)	95% CI	Year fixed effects	MSA^a^ fixed effects	Observations	MSAs	χ^2^
**5 miles of city hall**								
	Low	–0.65 (0.44)	–1.50 to 0.21	Yes	Yes	322	74	125.50 (7)
	High	0.63 (0.23)	0.17 to 1.09	Yes	Yes	406	74	345.94 (7)
**2 miles of city hall**								
	Low	–0.14 (0.56)	–1.24 to 0.96	Yes	Yes	317	71	108.53 (7)
	High	0.52 (0.24)	0.06 to 0.99	Yes	Yes	406	72	346.20 (7)

^a^MSA: Metropolitan Statistical Area.

## Discussion

### Principal Findings

We examined the association between ride-hailing platform entry and alcohol consumption. While prior work has tied ride-hailing to decreases in levels of drunk driving [13,15], the secondary implications of low-cost urban mobility have received scant attention. Results are nuanced. On one hand, ride-hailing is not associated with the number of people who consume alcohol in general (ie, the extensive margin). This is encouraging and suggests the presence of ride-hailing is not causing greater numbers of people to consume alcohol. The platform is, however, significantly associated with binge drinking in densely populated markets (ie, the intensive margin). This is cause for concern. Given the national and personal costs associated with binge drinking [32], these findings compound an already alarming national trend [33]. This suggests that although ride-hailing may reduce drunk driving, people update their behavior to exploit the assured mobility ride-hailing offers.

These findings suggest that Uber’s entry increases binge drinking by 4% in adult urban populations and point to valuable directions for future research. Prior work indicates that most binge drinking occurs among the young (aged 18 to 34 years) and is twice as common among men [25]. As ride-hailing associates with binge drinking, it is likely that the association would be concentrated in younger populations. However, this is speculative, and it is important to assess where behavioral changes are occurring. To the extent that means of viable travel are already established for younger groups, specifically when they are under the influence, it is plausible that these groups may also fail to exhibit changes in their behavior once Uber arrives. Ride-hailing may, instead, be associated with binge drinking increases among groups that are traditionally less at risk, by affording them greater mobility. It is also important to assess whether or not these differences manifest asymmetrically between men and women. Inasmuch as alcohol consumption has been associated with the incidence of sexual assault [12,34] and lesser infractions like property crime [8], the possible negative implications of Uber arrival for public safety through its relationship with binge drinking are important to consider. These services may also benefit public safety (eg, by affording a secure means of transport home late at night). Additional work is clearly needed to tease out whether ride-hailing services have a net beneficial or detrimental association with public safety measures.

### Limitations

This work is subject to certain limitations. Most notably, our estimates focus on the entry of ride-hailing services. The exact reasons that ride-hailing services significantly associate with increases in binge drinking in our sample are not altogether clear. This is a natural limitation of secondary data. Several possibilities exist. For example, recent work has noted that ride-hailing services can, in some instances, enhance access to public transit [23]. As such, the result we observe may be explained not just by ride-hailing services access but access in tandem with public transit options (which tend to be better in areas characterized by high population density).

Because we lack individual level data, the underlying associations are likely to be much larger for subpopulations that are most affected. One potentially valuable path forward, which our sample does not allow us to explore, are to consider effects across different subpopulations (eg, age, gender, and local area characteristics like educational composition and access to mass transit). In this context, where we to explore various sources of heterogeneity in a data-driven, exploratory manner, it would be necessary to implement stringent corrections for multiple corrections effectively reducing our already limited statistical power. Future work can look to address these aspects.

Our findings may be vulnerable to time-varying unobserved confounds (ie, unobserved factors) that might correlate over time with both Uber’s entry and binge drinking rates. That said, for this to be a concern, the dynamic confounds would need to vary systematically with Uber’s entry timing, which seems unlikely. Finally, our sample is potentially not representative, given the CDC’s application of sampling criteria in its administration of the BRFSS. The fact that the BRFSS does not report levels of drinking for all MSA-years does raise concerns of external validity. Yet, as the reported MSAs are almost uniformly larger and more densely populated, it is likely that the unreported MSAs would react in a way that is similar to the less densely populated MSAs reported in the sample. Still, future work is needed to tease out these dynamics robustly.

### Conclusion

Policy makers must consider the full range of public health consequences when regulating ride-hailing services and accordingly design appropriately nuanced interventions. While the observed drop in drunk driving is clearly beneficial, any causal association with increased binge drinking would be problematic. Two paths forward are thus evident. First, traditional health information campaigns have focused nearly exclusively on preventing people from getting behind the wheel. Drunk driving rates have been dropping for more than a decade, and this process may have been accelerated by ride-hailing. However, the evidence presented in this work indicates that messaging regarding alcohol consumption may need to be updated to address the growing problem of binge drinking. That is, while the information campaign against drunk driving should continue, our findings suggest that policy makers may instead wish to focus on the direct health effects of binge drinking. Second, it may be useful to partner with ride-hailing services to incentivize behavior through the platform. Beyond training drivers on how to appropriately manage and deal with intoxicated customers (eg, how to identify when they should be taken to hospital for medical care), opportunities exist to track excessive drinking at either the individual or local level (ie, behavioral surveillance) and incentivize more responsible behavior among citizens (ie, subsidized discounts for not drinking to excess). Critical to the success of these programs will be to ensure the platform is not imposing additional costs on binge drinkers, as this creates an incentive for them to return to driving under the influence.

We hope this work serves as a greater call to continue to explore the relationships between ride-hailing and public population health factors as well as the peer to peer sharing economy more broadly. Given that policy makers can only design effective interventions when presented with a full set of facts regarding the consequences of ride-hailing services, it is incumbent upon the research community to continue to provide rigorous insights, be they positive or negative, for practice or policy. Only in this way can we enable policy makers to implement steps that attenuate the negative aspects of ride-hailing and other digital platforms and amplify the positive aspects.

## Appendix

Multimedia Appendix 1 Uber entry schedule.

## Abbreviations

BRFSS: Behavioral Risk Factors Surveillance Survey
CDC: Centers for Disease Control and Prevention
MSA: Metropolitan Statistical Area
