# Supplementary material for: Ride-Hailing Services and Alcohol Consumption: Longitudinal Analysis
Source: J Med Internet Res. 2021 Jan 27;23(1):e15402. doi: 10.2196/15402 (PMC7875688; doi:10.2196/15402)
Supplement: Multimedia Appendix 1 [file jmir_v23i1e15402_app1.docx]

**Multimedia Appendix 1. Uber entry schedule.**

Below we provide the list of Uber entries and associated dates associated with MSAs in our panel (i.e., those captured as part of the BRFSS SMART dataset, specifically in regard to the “Binge Drinking” and “Any Drinking” questions). In each case, we record Uber entry based on the first appearance of any Uber offering in a location within the respective MSA (regardless of service format; Uber Black or Uber X).

| **Table A1. Uber Entry Schedule** | |
| --- | --- |
| **Uber Entry** | **Metropolitan Statistical Area (MSA) Description** |
| 5/31/10 | San Francisco-Redwood City-South San Francisco, CA |
| 5/31/10 | Oakland-Hayward-Berkeley, CA |
| 5/3/11 | New York-Jersey City-White Plains, NY-NJ |
| 5/3/11 | Newark, NJ-PA |
| 8/12/11 | Seattle-Bellevue-Everett, WA |
| 9/22/11 | Chicago-Naperville-Elgin, IL-IN-WI |
| 3/8/12 | Los Angeles-Long Beach-Anaheim, CA |
| 6/6/12 | Philadelphia, PA |
| 8/6/12 | San Diego-Carlsbad, CA |
| 8/24/12 | Atlanta-Sandy Springs-Roswell, GA |
| 9/7/12 | Denver-Aurora-Lakewood, CO |
| 9/9/12 | Boston, MA |
| 10/25/12 | Minneapolis-St. Paul-Bloomington, MN-WI |
| 11/1/12 | Phoenix-Mesa-Scottsdale, AZ |
| 1/3/13 | Dallas-Plano-Irving, TX |
| 2/1/13 | Reno, NV |
| 2/1/13 | Sacramento--Roseville--Arden-Arcade, CA |
| 3/28/13 | Detroit-Dearborn-Livonia, MI |
| 3/28/13 | Warren-Troy Farmington Hills, MI |
| 6/24/13 | Indianapolis-Carmel-Anderson, IN |
| 7/25/13 | San Jose-Sunnyvale-Santa Clara, CA |
| 8/8/13 | Washington-Arlington-Alexandria, DC-VA-MD-WV |
| 9/11/13 | Charlotte-Concord-Gastonia, NC-SC |
| 9/15/13 | Providence-Warwick, RI-MA |
| 10/5/13 | Tucson, AZ |
| 10/22/13 | Baltimore-Columbia-Towson, MD |
| 10/30/13 | Oklahoma City, OK |
| 11/13/13 | Atlantic City-Hammonton, NJ |
| 12/20/13 | Columbus, OH |
| 2/21/14 | Houston-The Woodlands-Sugar Land, TX |
| 3/6/14 | Madison, WI |
| 3/6/14 | Tallahassee, FL |
| 3/13/14 | Harrisburg-Carlisle, PA |
| 3/13/14 | Pittsburgh, PA |
| 3/19/14 | Cincinnati, OH-KY-IN |
| 3/27/14 | Tulsa, OK |
| 3/28/14 | San Antonio-New Braunfels, TX |
| 3/28/14 | Milwaukee-Waukesha-West Allis, WI |
| 4/3/14 | Riverside-San Bernardino-Ontario, CA |
| 4/8/14 | Cleveland-Elyria, OH |
| 4/11/14 | Tampa-St. Petersburg-Clearwater, FL |
| 4/24/14 | Louisville/Jefferson County, KY-IN |
| 4/24/14 | Hartford-West Hartford-East Hartford, CT |
| 5/1/14 | Virginia Beach-Norfolk-Newport News, VA-NC |
| 5/2/14 | Colorado Springs, CO |
| 5/5/14 | Jacksonville, FL |
| 5/5/14 | Omaha-Council Bluffs, NE-IA |
| 5/8/14 | Spokane-Spokane Valley, WA |
| 5/9/14 | Kansas City, MO-KS |
| 5/20/14 | Albuquerque, NM |
| 5/27/14 | Logan, UT-ID |
| 5/27/14 | Salt Lake City, UT |
| 6/4/14 | Miami-Fort Lauderdale-West Palm Beach, FL |
| 6/4/14 | Orlando-Kissimmee-Sanford, FL |
| 6/5/14 | Austin-Round Rock, TX |
| 6/12/14 | Honolulu, HI |
| 6/13/14 | Toledo, OH |
| 6/13/14 | Lexington-Fayette, KY |
| 6/26/14 | Raleigh, NC |
| 6/26/14 | Lubbock, TX |
| 6/26/14 | El Paso, TX |
| 7/4/14 | Lakeland-Winter Haven, FL |
| 7/10/14 | Columbia, SC |
| 7/10/14 | Myrtle Beach-Conway-North Myrtle Beach, SC-NC |
| 7/10/14 | Charleston-North Charleston, SC |
| 7/10/14 | Spartanburg, SC |
| 7/16/14 | Amarillo, TX |
| 7/24/14 | Lansing-East Lansing, MI |
| 8/4/14 | Greeley, CO |
| 8/6/14 | Richmond, VA |
| 8/13/14 | Grand Rapids-Wyoming, MI |
| 8/21/14 | Asheville, NC |
| 8/28/14 | Knoxville, TN |
| 8/28/14 | Wichita, KS |
| 8/28/14 | College Station-Bryan, TX |
| 8/28/14 | Gainesville, FL |
| 8/28/14 | Dayton, OH |
| 8/28/14 | Lincoln, NE |
| 8/29/14 | Fayetteville-Springdale-Rogers, AR-MO |
| 8/29/14 | Fort Smith, AR-OK |
| 8/30/14 | Lafayette, LA |
| 9/10/14 | St. Louis, MO-IL |
| 9/12/14 | Des Moines-West Des Moines, IA |
| 9/19/14 | New Orleans- |
| 10/2/14 | Portland-South Portland, ME |
| 10/2/14 | Boise City, ID |
| 10/9/14 | Burlington-South Burlington, VT |
| 10/11/14 | Akron, OH |
| 10/11/14 | Canton-Massillon, OH |
| 11/6/14 | Roanoke, VA |
| 11/6/14 | Little Rock-North Little Rock-Conway, AR |
| 11/13/14 | Chattanooga, TN-GA |
| 11/19/14 | Santa Fe, NM |
| 12/3/14 | Laconia NH |
| 12/4/14 | Cedar Rapids, IA |
| 12/4/14 | Memphis, TN-MS-AR |
| 12/4/14 | Cape Coral-Fort Myers, FL |
| 12/5/14 | North Port-Sarasota-Bradenton, FL |
| 12/5/14 | Portland-Vancouver-Hillsboro, OR-WA |
| 12/9/14 | Deltona-Daytona Beach-Ormond Beach, FL |
| 12/9/14 | Port St. Lucie, FL |
| 12/9/14 | Ocala, FL |
| 12/11/14 | Jackson, MS |
| 12/15/14 | Pensacola-Ferry Pass-Brent, FL |
| 1/30/15 | Allentown-Bethlehem-Easton, PA-NJ |
| 2/6/15 | Scranton--Wilkes-Barre--Hazleton, PA |
| 4/6/15 | Augusta-Richmond County, GA-SC |
| 4/23/15 | Topeka, KS |
| 4/24/15 | Springfield, MA |
| 4/30/15 | Kahului-Wailuku-Lahaina, HI |
| 5/11/15 | Fargo, ND-MN |
| 5/13/15 | Birmingham-Hoover, AL |
| 6/4/15 | Idaho Falls, ID |
| 6/4/15 | Coeur d'Alene, ID |
| 6/11/15 | Mobile, AL |
| 7/5/15 | Fort Wayne, IN |
| 7/21/15 | Davenport-Moline-Rock Island, IA-IL |
| 9/18/15 | Las Vegas-Henderson-Paradise, NV |
| 9/30/15 | Bellingham, WA |
| 11/11/15 | Cumberland, MD-WV |
| 11/19/15 | Baton Rouge, LA |
| 11/30/15 | Yakima, WA |
| 11/30/15 | Kennewick-Richland, WA |
| 1/6/16 | Montgomery, AL |
| 3/3/16 | Huntsville, AL |
| 3/21/16 | Lewiston-Auburn, ME |
| 3/22/16 | Bangor, ME |
| 3/22/16 | Great Falls, MT |
| 6/19/16 | Huntington-Ashland, WV-KY-OH |
| 6/19/16 | Charleston, WV |
| 6/23/16 | Youngstown-Warren-Boardman, OH-PA |
| 7/6/16 | Missoula, MT |
| 7/11/16 | Olympia-Tumwater, WA |
| 8/15/16 | Billings, MT |
| 8/16/16 | Tuscaloosa, AL |
| 1/10/17 | Albany-Schenectady-Troy, NY |
| 1/10/17 | Buffalo-Cheektowaga-Niagara Falls, NY |
| 1/10/17 | Glens Falls, NY |
| 1/10/17 | Rochester, NY |
| 1/10/17 | Syracuse, NY |
| 1/10/17 | Utica-Rome, NY |
| 1/10/17 | Binghamton, NY |
